# Supporting Child Development Through Parenting Interventions in Low- to Middle-Income Countries: An Updated Systematic Review

**DOI:** 10.3389/fpubh.2021.671988

**Published:** 2021-07-16

**Authors:** Linlin Zhang, Derrick Ssewanyana, Marie-Claude Martin, Stephen Lye, Greg Moran, Amina Abubakar, Kofi Marfo, Joyce Marangu, Kerrie Proulx, Tina Malti

**Affiliations:** ^1^Key Laboratory of Learning and Cognition, School of Psychology, Capital Normal University, Beijing, China; ^2^Alliance for Human Development, Lunenfeld-Tanenbaum Research Institute, Toronto, ON, Canada; ^3^Department of Psychology, Western University, London, ON, Canada; ^4^Institute for Human Development, Aga Khan University, Nairobi, Kenya; ^5^Centre for Child Development, Mental Health, and Policy, University of Toronto Mississauga, Mississauga, ON, Canada; ^6^Department of Psychology, University of Toronto, Toronto, ON, Canada

**Keywords:** parenting intervention, stimulation, early childhood development, low- and middle-income countries, systematic review

## Abstract

**Background:** Over 250 million children in low- and middle-income countries are at risk of not achieving their fullest developmental potential due to co-occurring risks such as poor nutrition and inadequate learning opportunities. Early intervention programs integrating the aspects of nurturing care, that is, good health, adequate nutrition, safety and security, responsive caregiving, and learning opportunities, may ameliorate against the negative impact of these adverse conditions.

**Methods:** This meta-analytic review updates the evidence base of parenting interventions comprising stimulation and responsive caregiving components on developmental outcomes for children under age 2 years in low- and middle-income countries. It also describes and assesses the moderation effects of population characteristics and implementation features on the intervention effectiveness. Studies were identified based on previous systematic reviews and an updated literature search in eight databases and the gray literature up to December 2020. A random-effect model was used to explore the pooled effect sizes accounted for by the intervention for developmental outcome of cognition, language, motor, and social-emotional capacities. Exploratory moderation analyses were also conducted.

**Results:** Twenty-one randomized controlled trials representing over 10,400 children from 12 low- and middle-income countries and regions across three continents (Africa, Latin America, and Asia) were identified. The interventions showed overall small-to-moderate effects on children's cognitive development (*ES* = 0.44; 95% CI = [0.30, 0.57]); language development (*ES* = 0.33; 95% CI = [0.18, 0.49]); and motor skills (*ES* = 0.21; 95% CI = [0.10, 0.32]). The overall effect on social-emotional development was non-significant (*ES* = 0.17; 95% CI = [−0.01, 0.34]). Effect sizes (ES) varied significantly across the studies. Parenting programs that targeted vulnerable groups, including rural communities and caregivers with lower education levels, had more significant effects on children's development. Group sessions (vs. individual visits) and high program dose (≥12 sessions) were also associated with stronger effects on child development. Further research is needed to determine the effectiveness of the workforce and training on programmatic outcomes.

**Conclusion:** The findings indicate that parenting interventions that encourage nurturing care are effective in improving the early development of children, especially among vulnerable populations. We discuss opportunities to strengthen the implementation of research-based parenting interventions in such contexts.

## Introduction

In low- and middle-income countries (LMICs), over 250 million children under age five are not achieving their developmental potential due to adverse living environments, such as chronic poverty, poor nutrition and sanitation, violence, and inadequate learning opportunities (1). As emphasized in multiple United Nations Sustainable Development Goals, child development has been recognized as playing a key role in the long-term development and well-being of the society at large (2–4).

The first few years of life are sensitive periods for brain and neural development, laying the foundation for long-term mental development (5–7). Specifically, there has been a push for the improved focus and need for investment in the first 1,000 days of life (which equates to the period from pregnancy to 2 years) (8). During this period, children are highly susceptible to environmental influences such as responsive parenting and cognitive stimulation. On the one hand, exposure to risk factors during this time can pose long-term and sometimes difficult-to-reverse detrimental impacts on children's developmental, educational, and health outcomes and productivity, ultimately incurring costs to society (9). Alternatively, this can also be a window for children to benefit from a nurturing caregiving home environment and thrive despite the adverse larger social environment (10).

Caregivers provide the primary environment in the early years of a child's life and are the entry point of many interventions supporting child development (11, 12). Such interventions include parenting programs, which are defined as interventions or services aimed at improving parenting interactions, behaviors, knowledge, attitudes, practices and beliefs (10). While earlier parenting interventions had a heavy emphasis on child health and nutrition, more and more research has recognized the importance and potential of psychosocial stimulation, especially given the shift from surviving and promoting physical growth to thriving and nurturing mental development (1). According to the World Health Organization, psychosocial stimulation consists of physical and emotional stimulation which are both aimed at facilitating children's cognitive, emotional, social and language development (13). Emotional stimulation comprises the expression of affection and warmth to a child in ways that are consistent with cultural norms, responding to the needs of the child in a timely manner, encouraging verbal and non-verbal communication between caregiver and the child, and praising or showing appreciation when the child manages to do something (13). Physical stimulation involves providing the child with opportunities for adequate sensory experiences through playing together with the child and providing them with age-appropriate play materials as well as, providing meaning to the child's physical world for example, by helping the child to name, count, and compare objects (13). The concept of psychosocial stimulation is also captured in the Nurturing Care Framework under its components of “responsive caregiving” and “opportunities for early learning” (14). Responsive caregiving entails propensity on the part of caregivers to notice, understand and respond to their child's cues in an appropriate and timely manner. Responsive care also creates opportunities for early learning, which refers to any opportunity for a baby or child to interact with objects, people, and place in their environment (14). Responsive feeding is also embedded in the concept of responsive caregiving and involves practices such as minimizing distractions during meals, feeding slowly and patiently, talking to a child with eye-to-eye contact during feeding, and introducing different food combinations (textures, tastes) at an appropriate age (15, 16). Overall, psychosocial stimulation interventions/programs train caregivers on how to support their children's development through responsive and sensitive caregiver-child interactions (e.g., age-appropriate play, telling stories, responsive feeding, exploring picture books, praising, cuddling among others). Noteworthy, psychosocial stimulation interventions are increasingly being integrated into other maternal and child health programs like nutrition, sanitation, and cash transfer programs (17, 18).

Along this line, several psychosocial stimulation or responsive care interventions have been implemented for children younger than 2 to 3 years of age in LMICs (10, 17, 19–22). A recent review of 75 parenting programs, which included 14 studies (12 from LMICs) on psychosocial stimulation or responsive care, found that in LMICs, these interventions had significant effects on cognitive development [ES = 0.49, CI (0.27–0.72)], language skills [ES = 0.43, CI (0.11–0.76)], and on motor skills development [ES = 0.39, CI (0.13–0.65)] in children below 5 years (17). Previous reviews have also corroborated the findings of moderate positive effects of parenting interventions with psychosocial stimulation aspects on language outcomes (21, 22), cognitive skills (10, 21, 22), motor development (10) and psycho-social skills (10) of young children. Another most recent review consistently reports the positive benefits of these interventions on cognitive development but notes that these benefits are only sustained for a short-term (1–3 years) duration (20). These results suggest that caring and stimulating environments with opportunities to play and communicate do play a significant role in child development outcomes.

Despite the promising effect of parenting interventions that include psychosocial stimulation or responsive care on developmental outcomes of children in low-resource settings, the key factors contributing to and barriers preventing the success of the interventions are still not well-understood. It has been proposed that the intervention and implementation processes, such as the mode of delivery (i.e., home visits, group sessions, or clinic appointments) (1, 4–6); the sensitivity of the measures utilized to assess child developmental outcomes (3, 4); the frequency of sessions and overall duration of the intervention (2, 5); the curriculum characteristics, for example, the form of behavior change techniques used (1, 2, 4, 6); the characteristics of the delivery agents (e.g., level and quality of training, being a part of existing service delivery systems) (2, 4); and the fidelity and quality control measures in place (2, 5) are likely important factors besides the content that account for the level of effectiveness and sustainability of the intervention. The characteristics of the target population, including caregivers' education status, household socioeconomic status, characteristics of children (e.g., age at enrolment), and caregivers' study setting (e.g., rural or urban) are also outlined among potential sources of variation in intervention effects (17, 19–21). Thus, researchers have increasingly expressed a need for an improved understanding of the common mechanisms that drive sustained parenting intervention treatment gains (20).

Although there has been consensus that these underlying factors require careful inspection, only the meta-analysis by Aboud and Yousafzai (2015) included some form of moderation analysis to inspect variation attributable to some of these factors. They found that the weighted mean of 10 effects sizes was improved (from d = 0.324 to 0.592) upon stratification based on seven interventions which utilized group sessions with some home visits, indicating the potential advantage of using a mixed approach (home visits with group sessions) over interventions with only home visits or clinic visits separately (21). They also noted that the implementation of parenting interventions requires a structured curriculum with sufficient dosage but not overloading messages, a format of delivery, well-trained and supervised personnel. However, they found that only 5 of the 21 studies in their review reported on fidelity to the intended program (21). Generally, most of the existing reviews descriptively summarize a few intervention/implementation factors with mixed depths of discussion (mainly briefly) about their potential implications for the findings and future work on parenting interventions and policy/decision-making. Thus, how intervention effects vary across other implementation delivery methods and sample characteristics are still not well-understood.

### The Present Study

The current systematic meta-analytic review builds upon previous reviews (10, 17, 19–22) and aims to extend the knowledge of parenting interventions and their developmental outcomes, specifically interventions with the components of psychosocial stimulation or responsive care for children under the age of 2 years in low- and middle-income countries. The specific objectives are: (i) to update the existing literature of the effectiveness of parenting interventions with components of psychosocial stimulation or responsive caregiving on cognitive, language, motor, and socio-emotional skills development of children under the age of 2 years in LMICs; and (ii) to describe and assess the moderation effects of implementation and sample characteristics on the outcomes of interventions with the components of psychosocial stimulation or responsive care. We, therefore, conducted exploratory moderation analyses, whenever possible, on intervention effect sizes across select study characteristics that were identified to be key in successful interventions (21) and also described a variety of other implementation aspects.

## Methods

### Literature Search

Parenting interventions that include psychosocial stimulation or responsive care of children under age two in low- and middle-income countries were extracted from previous systematic reviews on this topic. At the inception of this work, the available literature dated till 04/2015 (10, 21, 22). We conducted an updated literature search from 05/2015 to 10/2020. Our systematic review followed the guidelines for preferred reporting items for systematic reviews and meta-analyses (PRISMA) (23). The updated literature search was conducted in eight electronic databases of PubMed, EMBASE, CINAHL, Scopus, PsycInfo, ERIC, ProQuest Dissertation & These Global, and EconLit. Additionally, gray literature was searched in the OAIster and OpenGrey databases and institutional electronic resources (e.g., websites of UNICEF, WHO, The World Bank). Reference tracking and hand-searching were also conducted to identify any relevant materials that could have been missed during the indexing process, as well as any additional relevant articles published between 11/2020 and 12/2020. The search strategy comprised a combination of free terms or keywords on participants (e.g., child, toddler, infant), types of intervention (e.g., attachment, stimulation, psychosocial), and types of outcomes (e.g., development, cognitive, language, social, motor) combined with Boolean operators of “OR” and “AND” (see Supplementary Table 1).

### Inclusion and Exclusion Criteria

Articles were screened by title, abstract and full text for eligibility by trained undergraduate psychology research assistants, the first author and a co-author author (DS). Studies were included if they met the following criteria: (1) the intervention focused on improving caregiving quality through individual or group-based training on responsive, stimulating, and sensitive caregiver-child interactions (e.g., age-appropriate play, telling stories, responsive feeding, exploring picture books, praising, cuddling among others); (2) the study enrolled children with an average age below 24 months; (3) the study reported quantitative findings on at least one of the childhood developmental outcomes of cognitive, motor, social-emotional capacities, and language skills; (4) the intervention was implemented in low and middle-income countries; and (5) the study used randomized controlled design. Studies were excluded if they only enrolled children or caregivers with selected pre-existing health risks such as preterm infants or infants of mothers with HIV and if they did not report quantitative findings on any of the childhood developmental outcomes.

### Data Extraction and Quality Assessment

The information extracted from the eligible studies included: study characteristics (e.g., country, study design, period of study implementation, enrolment criteria, caregiver age), intervention and implementation features (e.g., curriculum used, delivery mode, dosage, intervention facilitators, characteristics of delivery agents, capacity building, formative research and quality assurance aspects), effect sizes on child developmental outcomes and assessment instruments. Quality assessment of the studies in this review was conducted following the Cochrane Collaboration Risk of Bias guidelines (24). Specifically, the studies were rated on the potential of bias (high, low, unclear) in the following domains: random sequence generation, allocation concealment, blinding of participants and personnel, blinding of outcome assessors, completeness of outcome data, and selectivity in reporting. Ratings of risks of bias were not used as an exclusion criterion (see Supplementary Table 2). Both the extraction and risk of bias assessment were conducted independently by the first author, a co-author (DS) and a trained graduate student using structured coding spreadsheets and discrepancies were resolved through discussion and consensus.

### Outcomes

This review focused on the outcomes in the four childhood developmental domains of cognitive, language, motor and social-emotional capacities, assessed at the first post-intervention period. The cognitive outcomes included broad cognitive processing such as sensory-perceptual skills, problems solving, memory (25), which are often captured collectively as scores on scales like the Bayley II Mental Developmental Index (26), the Bayley-III cognitive scale (27), and Performance subscale of the Griffiths Scales of Child Development (28). Language outcomes included communication skills such as receptive and expressive language (29). Motor outcomes included gross locomotor and fine motor skills such as hand and eye coordination (30). Social-emotional outcomes included a broad range of social and emotional capacities. Specifically, these are adaptive behaviors either collectively reported such as personal-social development skills on the Ages and Stages Questionnaire, or separately reported such as responsiveness to examiner, emotional tone, cooperation, and emotional vocalization using Wolke scale (31) and modified behavior rating scales (32, 33).

### Data Analysis

Data were synthesized both quantitatively and narratively. The effect sizes (ESs) for each of the four domains (cognitive, language, motor, and social-emotional capacities) were calculated as the standardized mean difference between the intervention and control groups at post-test using pooled standard deviations and weighted by the inverse variance method (Hedge's g) (34, 35). When the data reported in a study was not sufficient to compute the Hedge's g, we instead narratively summarized the effects without including the reported measure of effect in the quantitative pooled effects analysis. For studies with multiple outcomes within one category (e.g., expressive and receptive language), the effect sizes were aggregated under the assumption that the outcomes were moderately correlated (*r* = 0.50) when actual correlations were not available (36). For studies with multiple intervention or control groups, effect sizes were combined across the groups when possible following the procedures described in chapters 23–25 of the handbook by Borenstein et al. (36). Specifically, for intervention groups, we prioritized effect sizes from arms of a factorial randomized controlled trial which comprised the intervention package specifically focused on the responsive, stimulating, and sensitive caregiver-child interactions components. For example, if a study had four arms of “psychosocial stimulation alone,” “nutrition supplementation alone,” “psychosocial stimulation and nutrition supplementation,” and “control (routine care)” we focused on effect sizes from the “psychosocial stimulation alone” and the “control (routine care)” arms. Effect sizes were analyzed with a random-effect model to explore the estimates accounted for by the intervention on each of the four developmental outcomes. The random-effect model was fitted using the meta-analysis command “metan” in STATA 15 software package (37). The variation in effect size attributable to heterogeneity was evaluated with the *Q* statistic and the *I*^2^ statistic of the DerSimonian and Laird method (38).

Exploratory moderation analyses were conducted to examine whether the effect sizes varied significantly according to study characteristics. Sample characteristics included the context of the sample (0 = *rural*, 1 = *urban or peri-urban*), the mean age of children at enrollment (0 = *less than 12 months*, 1 = *equal to or greater than 12 months*), average levels of maternal education (0 = *lower than 6 years or over 50% did not complete primary education*, 1 = *greater than 6 years or over 50% completed primary and higher education*). Implementation characteristics included mode of delivery (0 = *individual visits*, 1 = primarily *group sessions or group sessions combined with individual visits*), average dosage of delivery (0 = *2 times or less per month*, 1 = *more than 2 times per month*), total number of sessions (0 = *less than 12 sessions*, 1 = *12 or more sessions*), duration of intervention (0 = *less than 12 months*, 1 = *12 months or longer*), characteristics of delivery agents (*0* = *trained paraprofessionals with previous experience in the healthcare system, 1* = *trained paraprofessionals without previous experience in the healthcare system, 2* = *mixed paraprofessionals and/or health professionals*) and duration of training of delivery agents (*0* = *2 weeks or more, 1* = *less than 2 weeks, 2* = *details not reported*).

Narrative synthesis involved a summary (both in text and table format) of risk of bias assessment, study characteristics (e.g., country, year of implementation), curriculum utilized, tools utilized to assess developmental outcomes, capacity building of intervention staff, quality control and fidelity aspects and formative research components of the interventions.

## Results

### Study and Sample Characteristics

**Figure 1:**
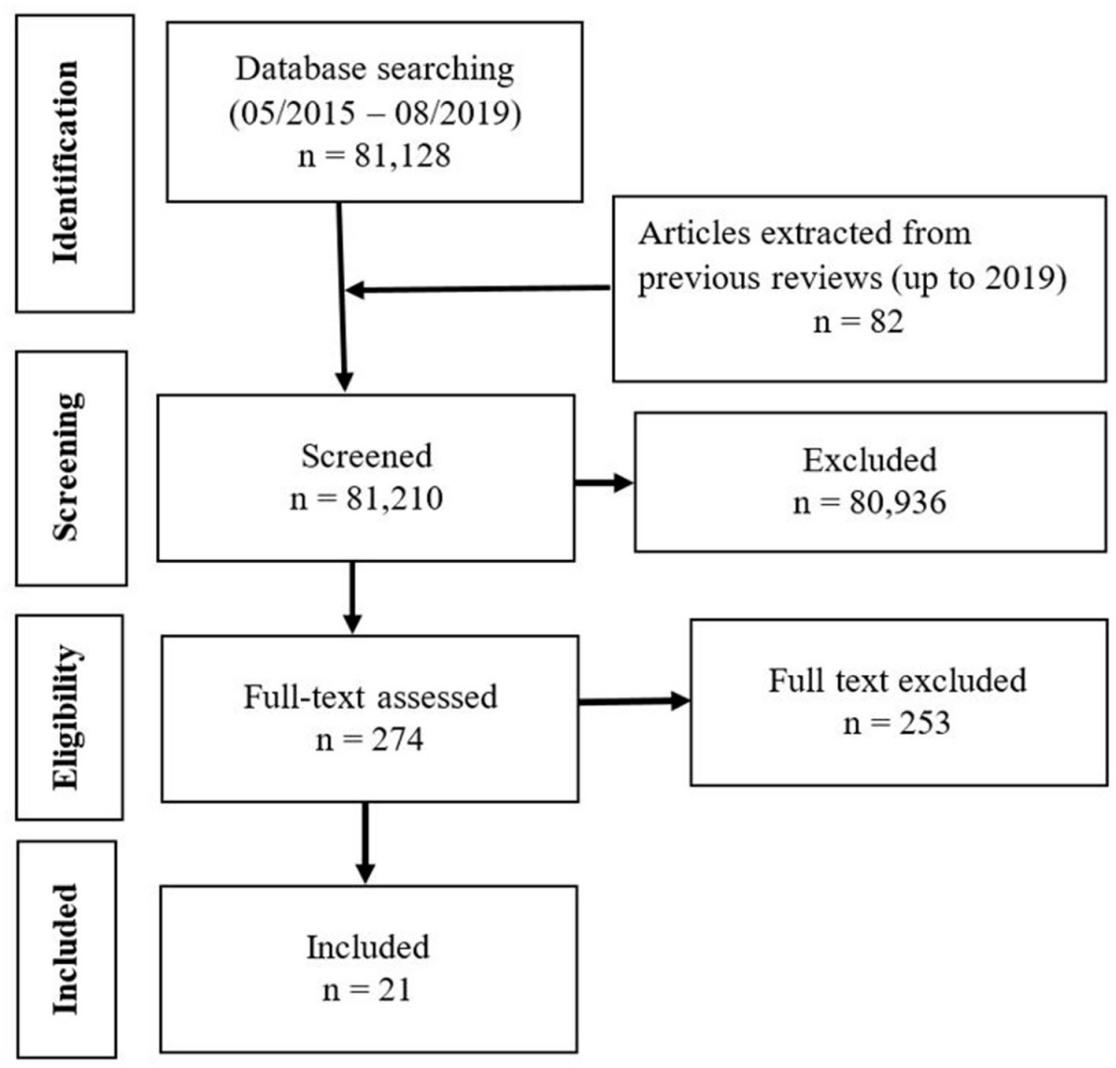
Flow chart showing the selection of studies.

Twenty-one studies were identified from a total of 81,210 records from the database search and previous reviews based on the eligibility criteria (see Figure 1 for the flow chart). Of the 21 eligible studies, 16 of them (39–54) were previously captured in four previous reviews (17, 20–22) assessing the effects of parenting interventions with the components of psychosocial stimulation or responsive care on early child neurodevelopmental outcomes. This present review includes 5 additional studies (55–59). Common reasons for exclusion include the use of a non-RCT design, study conducted outside a low-and-middle-income country context, a single focus on nutrition supplementation or education, the enrollment of predominantly children over 24 months, and the absence of child developmental outcomes (e.g., a focus on child physical growth or parent-child interactions).

The study and sample characteristics are summarized in Table 1. Of the 21 eligible studies, seven were conducted in the African region (42, 46, 49, 55–58), and their implementation period was between 2013 and 2019. There were four eligible studies from the Caribbean region (41, 50–52), implemented between 1999 and 2012; however, two of these studies did not report the implementation dates (50, 51). Both studies from the Latin American region were from Colombia, conducted between 2010 and 2016 (40, 59). Eight studies were conducted in Southern and South Eastern Asia (39, 43–45, 47, 48, 53, 54). The intervention period of the Asian studies was between 2000 and 2012; however, 3 of these (44, 45, 47) did not report the implementation periods. Over 10,400 children and families were enrolled in these 21 studies, with sample sizes ranging from 41 to 1,381 per study. Among these studies, 13 were conducted in rural areas and seven were in urban or peri-urban areas (1 unidentified). Children's age at enrollment ranged from pre-birth (i.e., third trimester of pregnancy) to 36 months with a mean below 12 months among 10 studies (39, 41, 42, 47, 49, 52–54, 58, 59) and between 12 to 24 months for the other 10 studies. Enrollment age was missing in one of the studies (55). Maternal education of the study participants was below primary level (mean <6 years or > 50% did not complete primary education) in 13 interventions (of note, almost all in rural contexts) and above the primary level in 7 interventions (40, 41, 51, 52, 56, 58, 59); which were mostly from the urban context. One of the studies did not report the maternal education level (50).

**Table 1:** Study and sample characteristics.

**Study (first author)**	**Context**	**Design**	**Year of intervention**	**Enrollment criteria**	**Average child age at baseline**	**Average maternal education**
Abimpaye (55)	Rwanda	Cluster RCT	2015-2016	Children aged 6 to 24 months	Not reported	96% primary and below
Aboud (48)	Rural Bangladesh	Cluster RCT	2008	Children aged 8-20 months from poor and very poor families excluding children who had disabilities and children who had not started complementary feeding	13–15 months	4–6 years
Aboud (54)	Rural and peri-urban Bangladesh	Cluster RCT	2011	Children aged 4–14 months excluding twins and severely sick and disabled children	6–11 months	5–6 years
Attanasio (40)	Urban Colombia	Cluster RCT	2010-2011	Children aged 12–24 months from the poorest 20% families who were enrolled in the Familias en Accion cash transfer program	18 months	7–8 years
Attanasio (59)	Rural Colombia	Cluster RCT	2014-2016	Children under 12 months	5–6 months	8–9 years
Barnhart (57)	Rwanda	Cluster RCT	2014-2015	Children aged 6 to 36 months	23.5 months	97% primary and below
Chang (41)	Urban Jamaica, Antigua, St. Lucia	Cluster RCT	2011-2012	Children aged 6–8 weeks from predominantly lower and lower-middle income families excluding preterm infants, multiple births, or infants receiving special care after birth	1–2 months	10 years
Gardner (50)	Urban Jamaica	Cluster RCT	not reported	Undernourished children aged 9–30 months excluding twins or children with physical or mental impairments	19 months	Not reported
Hamadani (43)	Rural Bangladesh	Cluster RCT	2000-2002	Undernourished children aged 6–24 months excluding children with developmental problems	15 months	52–57% less than 5 years
Jin (53)	Rural China	RCT	2003	Children aged 0–24 months from impoverished villages excluding those with a history of complicated delivery, significant medical treatment, or acute or chronic illness	10 months	22–36% illiterate
Luoto (56)	Kenya	Cluster RCT	2018-2019	children aged 6–24 months	14 months	8.8 years
Muhoozi (42)	Rural Uganda	Cluster RCT	2014	Children aged 6–8 months excluding those with congenital malformations, physical disorder, or mental illness	7 months	4.9 years
Murray (58)	Peri-urban South Africa	RCT	Not reported	Pregnant women in 3rd trimester	0 month	28–30% less than 6 years
Nahar (44)	Urban Bangladesh	RCT	Not reported	Severely malnourished children aged 6–24 months without acute infections	12–13 months	3–4 years
Powell (51)	Urban Jamaica	Cluster RCT	Not reported	Undernourished children aged 9–30 months without chronic disease and disability	18–19 months	38–43% completed high school
Rockers (49)	Rural Zambia	Cluster RCT	2014-2015	Children aged 6–12 months with caregivers aged 15 years and older	8–9 months	55% did not complete primary school
Singla (46)	Rural Uganda	Cluster RCT	2013	Children aged 12–36 months	22 months	4 years
Tofail (45)	Rural Bangladesh	Cluster RCT	Not reported	Children aged 6–24 months with and without iron deficiency anemia (IDA) excluding twins and children diagnosed congenital anomalies	15–16 months	5–6 years
Vazir (47)	Rural India	Cluster RCT	Not reported	Pregnant women in 3rd trimester	3 months	68–75% primary school or illiterate
Walker (52)	Urban Jamaica	RCT	1999	Low birth weight (LBW) infants at birth with maternal education below 3 secondary level examination passes excluding twins, those with congenital abnormalities, receiving special care nursey, and HIV positive mothers	0 month	3–9% primary and below
Yousafzai (39)	Rural Pakistan	Cluster RCT	2009-2012	Children aged 0-2.5 months without signs of severe impairments	0–1 months	2–3 years

### Assessment of Child Outcomes

All the seven studies in Africa reported cognitive outcomes, while five reported language skills (42, 46, 55–57) and 4 reported on social-emotional capacities and motor skills (42, 55–57). All four studies from the Caribbean reported cognitive, motor and language outcomes, but none reported socio-emotional data. Both studies in Colombia reported cognitive, motor and language outcomes. Dimensions of social-emotional development were only reported in one study in Colombia (59). Four studies in Asia reported on language (39, 48, 53, 54) and social-emotional outcomes (43, 45, 47, 53), while 5 reported on motor skills (43–45, 47, 53). Only one study did not report cognitive outcomes (48).

Assessment measures of child outcomes are summarized in Table 2. Twelve of the studies that assessed cognitive development used the Mental Development Index or the Cognition subscale of the Bayley Scales of Infant Development (Bayley II or Bayley III) (26, 27) and 4 used the Performance subscale of the Griffiths Scales of Child Development (28). The most frequently utilized tools for assessment of language development were the Receptive and Expressive Language subscales of the Bayley Scales (reported in 8 studies) and the Hearing and Speech subscale of the Griffiths Scales (reported in 4 studies). Similarly, the majority of studies on motor development utilized the Psychomotor or fine motor subscales of the Bayley Scales and the Hand and Eye Coordination subscale of the Griffiths Scales. The parents' report on Ages and Stages (60) (used in 4 studies) and the Behavior rating scales (in 2 studies) were the most commonly utilized tools in assessing social-emotional development.

**Table 2:** Assessment tools for developmental outcomes.

**Study**	**Cognitive**	**Language**	**Motor**	**Social-emotional**
Abimpaye (55)	ASQ	ASQ	ASQ	ASQ
Aboud (48)	—	Bayley III^a^	—	—
Aboud (54)	Bayley III	Bayley III	—	—
Attanasio (40)	Bayley III	Bayley III	Bayley III	—
Attanasio (59)	Bayley III	Bayley III	Bayley III	ASQ: SE
Barnhart (57)	ASQ	Malawi Developmental Assessment Tool, ASQ	Malawi Developmental Assessment Tool, ASQ	ASQ
Chang (41)	Griffiths	Griffiths	Griffiths	—
Gardner (50)	Griffiths	Griffiths	Griffiths	—
Hamadani (43)	Bayley II	—	Bayley II	Behavior ratings
Jin (53)	Gesell Schedule	Gesell Schedule	Gesell Schedule	Gesell Schedule
Luoto (56)	Bayley III	Bayley III	—	Wolke Scale
Muhoozi (42)	Bayley III	Bayley III	Bayley III	ASQ
Murray (58)	Bayley II	—	—	—
Nahar (44)	Bayley II	—	Bayley II	—
Powell (51)	Griffiths	Griffiths	Griffiths	—
Rockers (49)	Saving Brains	—	Saving Brains	—
Singla (46)	Bayley III	Bayley III	—	—
Tofail (45)	Bayley II	—	Bayley II	Behavior ratings
Vazir (47)	Bayley II	—	Bayley II	—
Walker (52)	Griffiths	Griffiths	Griffiths	—
Yousafzai (39)	Bayley III	Bayley III	Bayley III	Bayley III

*ASQ, Ages and Stages Questionnaire; ASQ: SE, Ages and Stages: Social-Emotional; Bayley III, Bayley Scales of Infant and Toddler Development III; Griffiths, Griffiths Mental Development Scales; Gesell Schedule, Gesell Development Schedules; Saving Brains, Saving Brains Early Child Development Scale;*

a *This study used 11 items and a different scoring system from the Bayley Language subscale*.

### Effect Sizes on Child Outcomes

#### Pooled Effect Sizes and Heterogeneity

**Figure 2:**
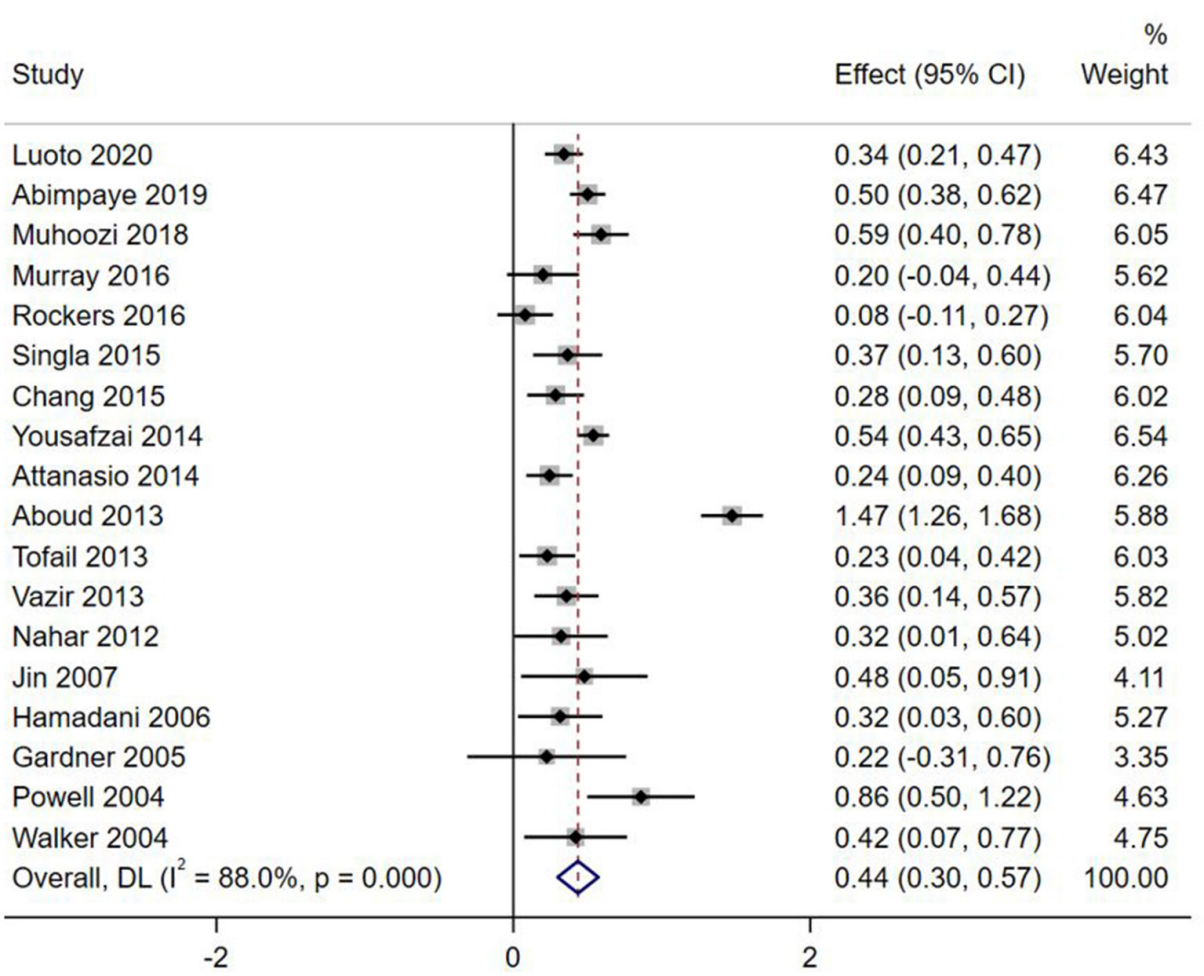
Effect sizes on Cognitive outcomes.

**Figure 3:**
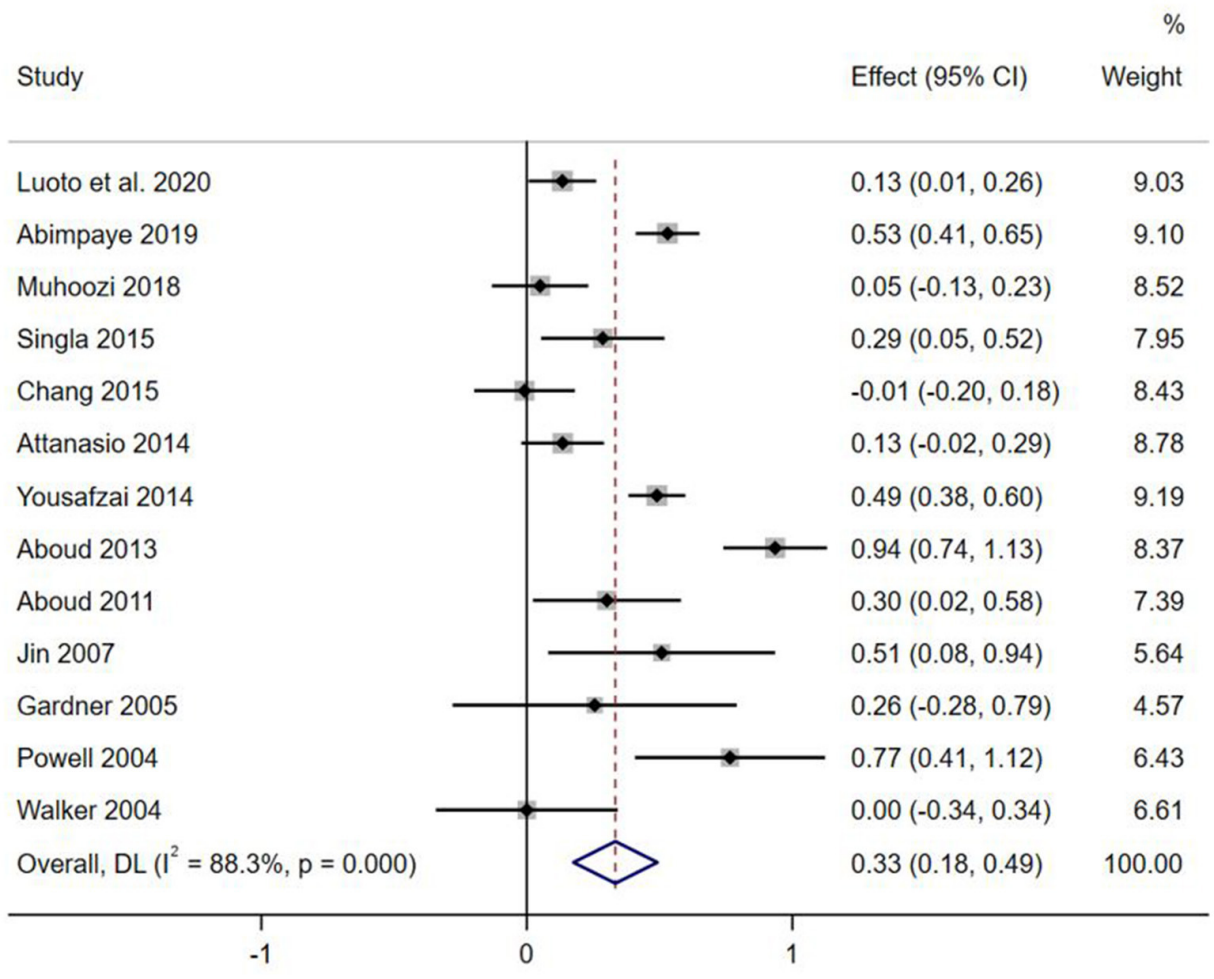
Effect sizes on Language outcomes.

**Figure 4:**
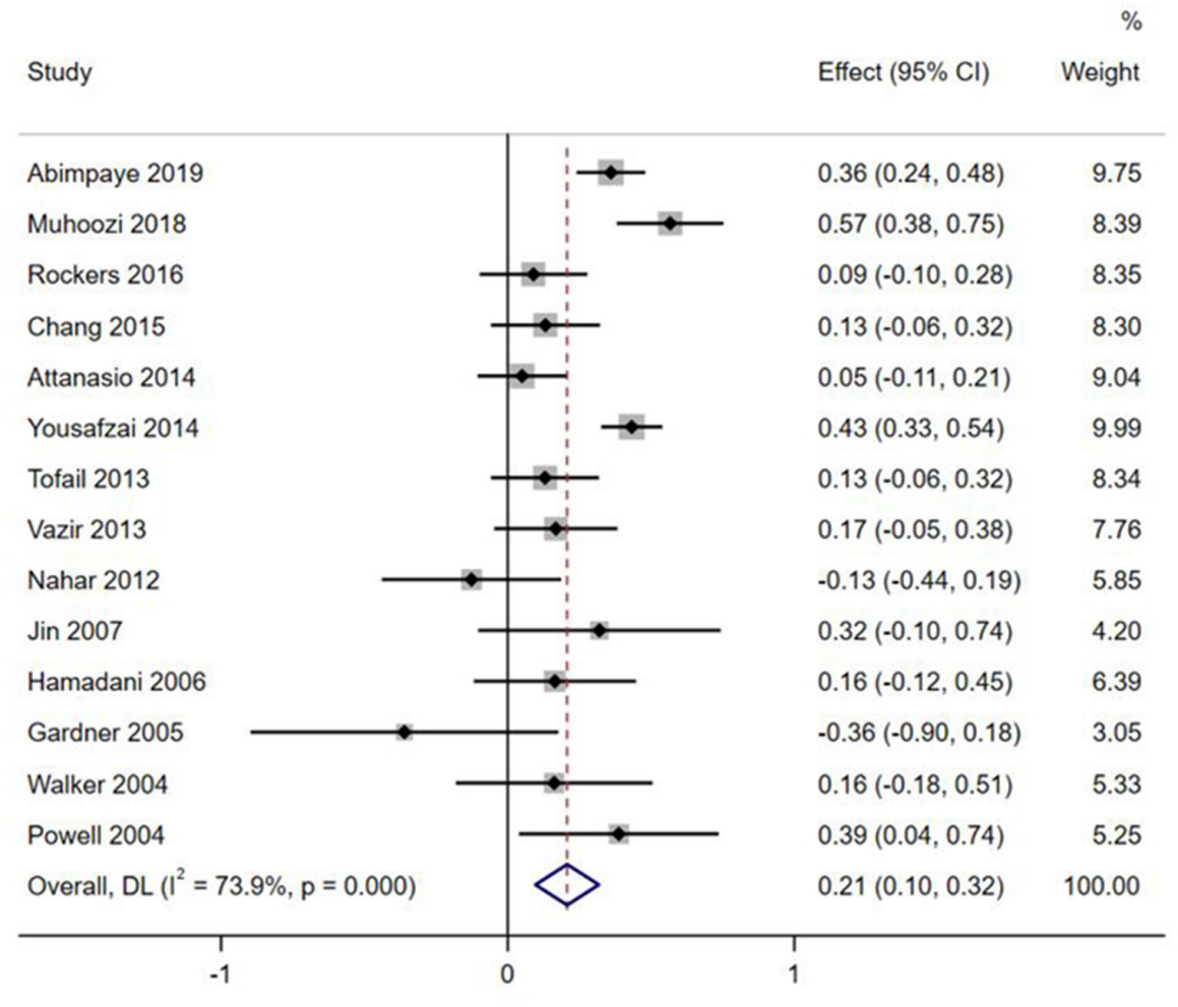
Effect sizes on Motor outcomes.

**Figure 5:**
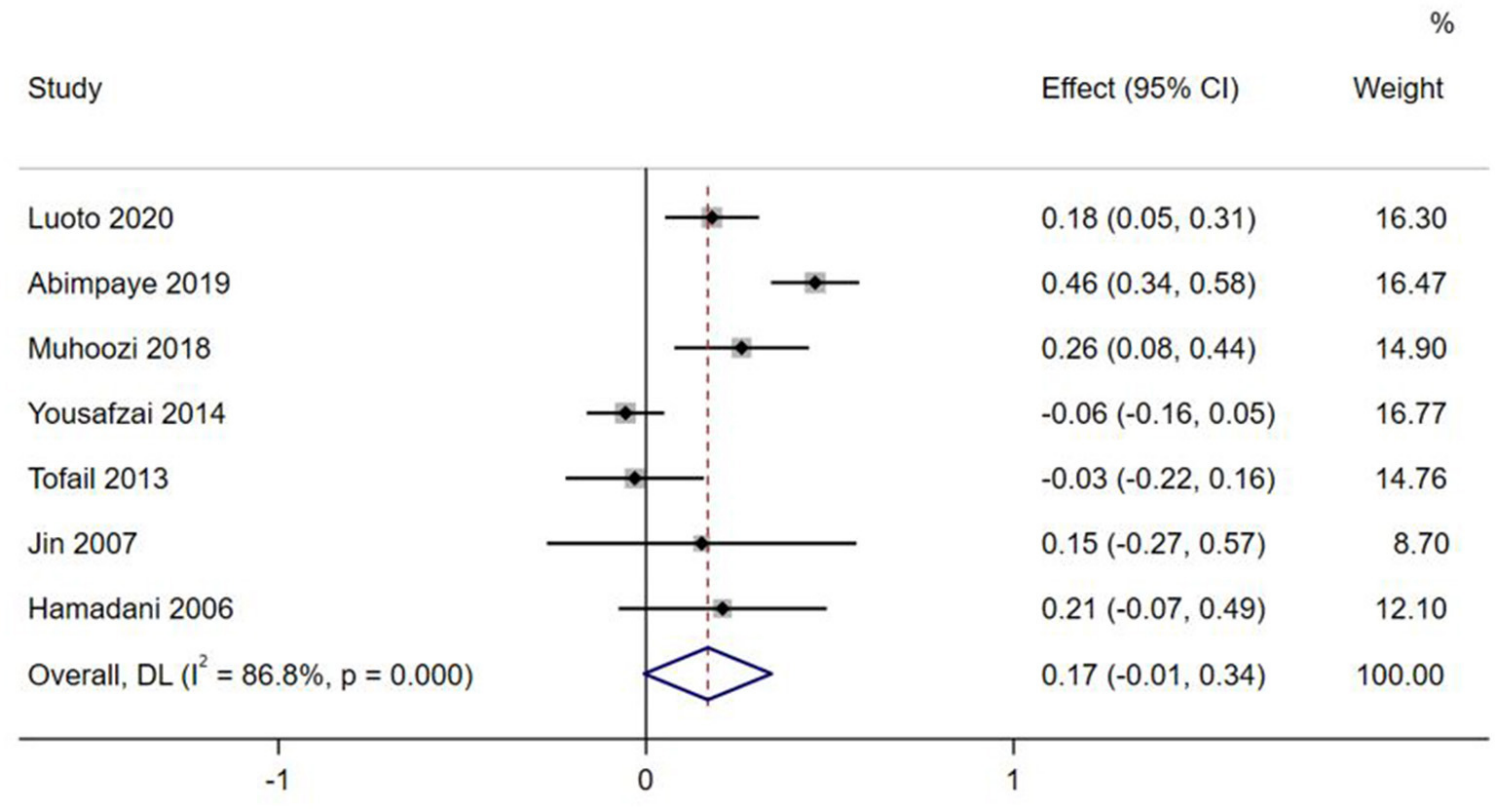
Effect sizes on Socio-emotional outcomes.

Effect sizes and forest plots of the individual studies are presented in Figures 2–5. The pooled average effect sizes are 0.44 on cognitive development (*n* = 18; 95% CI = [0.30, 0.57]), 0.33 on language development (*n* = 13; 95% CI = [0.18, 0.49]), 0.21 on motor development (*n* = 14; 95% CI = [0.10, 0.32]), and 0.17 on social-emotional development (*n* = 7; 95% CI = [−0.01, 0.34]). The effect sizes varied significantly across studies on cognitive development (*Q* = 141.99, *df* = 17, *p* < 0.001, *I*^2^ = 88.0), language development (*Q* = 102.39, *df* = 12, *p* < 0.001, *I*^2^ = 88.3), motor development (*Q* = 49.87, *df* = 13, *p* < 0.001, *I*^2^ = 73.9), and social-emotional development (*Q* = 45.33, *df* = 6, *p* < 0.001, *I*^2^ =86.8).

We did not include two studies in the meta-analysis as the data reported in both were not sufficient to compute the Hedge's g (57, 59).

### Intervention Characteristics

The intervention and implementation characteristics are narratively summarized in Table 3 and in the Supplementary Table 2.

**Table 3:** Intervention and implementation characteristics.

**Study**	**Intervention and Control groups used in the analyses**	**Intervention Arm**				**Control Arm**
		**Curriculum**	**Mode of delivery**	**Dosage and duration**	**Facilitator**	
Abimpaye (55)	Full touch vs. Control N_int_ = 486 N_ctrl_ = 479	First Steps parenting program with curricula around responsive caring and bonding, playful learning, care of physical health, and access to and use of baby books.	Group sessions	Weekly group sessions for 17 weeks	Local volunteers with at least 9-year education	No intervention except for the book seller activities that were run in both control and intervention sites
Aboud (48)	RFS vs. Control N_int_ = 99 N_ctrl_ = 101	Responsive parenting (feeding and stimulation) added onto the regular program (see details in the control arm).	Group sessions	5 weekly group sessions for 1-2 months plus 1 booster session 5 months after across 7 months	Young women from the village with a grade 9 education	The regular program provides 12 informational sessions delivered by community health workers on health, nutrition, and child development for 7 months.
Aboud (54)	Intervention vs. Control *combined younger and older groups* N_int_ = 221 N_ctrl_ = 226	Parenting practices related to health, nutrition, communication, and play.	Group sessions for one subgroup; individual visits for another subgroup.	14 group sessions fortnightly for 4 months and monthly for 6 months across 10 months; 1 to 5 visits of 10-min counseling at home and clinics.	Young women from the village with grade 10 education for group sessions; family welfare assistants for individual counseling.	Government standard care by family welfare assistants on feeding and hygiene.
Attanasio (40)	Stimulation (w/o supplementation) vs. no stimulation (control) N_int_ = 318 N_ctrl_ = 318	Play activities using low cost or homemade toys, picture books, and form boards adapted from the Jamaica Home Visiting program.	Home visits	78 weekly home visits for 18 months	Community mother leaders	Micronutrient supplementation for supplementation control; unclear for regular control
Attanasio (59)	Intervention vs. Control N_int_ = 628 N_ctrl_ = 707	Play activities adapted from the Jamaica Home Visiting program and messages on feeding and nutrition plus nutritional supplement	Group sessions and home visits	24 weekly group meetings and 24 monthly home visits across an average of 45 weeks	Women in the community with a high school degree	Government program which was also ran in intervention groups
Barnhart (57)	Intervention vs. Control N_int_ = 19 N_ctrl_ = 22	12 modules on: children's development, nutrition, health, and hygiene; coaching parents on responsive parenting and “serve and return” interactions; reducing violence; strengthening parental problem solving skills and social support; promoting early language learning and school readiness	Home visits	Weekly (60–90 min of 12 modules) for 12–16 weeks	Community Based volunteers with ability to read, write, and count in Kinyarwanda and committed to young children and family values	Only Social protection (cash-for-work opportunities and direct support via cash transfers)
Chang (41)	Intervention vs. Control N_int_ = 216 N_ctrl_ = 210	Messages on topics of love, responding and comforting, talking to children, praise, using bath time to play and learn, looking at books, simple toys to make, drawing and games, and puzzles shown in short films with discussion, demonstration, and practice afterward.	Group sessions at the clinic	5 group sessions during routine visits at the health center at 3, 6, 9, 12, and 18 months of age across 16 months	Community health workers	Routine care at the clinic
Gardner (50)	Stimulation vs. no stimulation (control) N_int_ = 21 N_ctrl_ = 38	Mother-child play activities to stimulate cognitive, language, fine motor, and problem-solving skills based on the Jamaica Home Visiting program.	Home visits	24 weekly home visits for 6 months	Community health workers	Routine care or placebo delivered by community health workers weekly
Hamadani (43)	Intervention vs. Control N_int_ = 92 N_ctrl_ = 101	Play activities emphasizing the importance of praising children, giving positive feedback, chatting with them, labeling things in the environment, and discouraging punishment with low-cost toys and books based on the Jamaica Home Visiting program added to the nutrition program	Group sessions & home visits	~44 group meetings weekly for 10 months and bi-weekly for 2 months & ~80 home visits twice weekly for 8 months and weekly for 4 months across an overall duration of 12 months	Literate women from the village	Nutritional surveillance and supplementation program
Jin (53)	Intervention vs. Control N_int_ = 45 N_ctrl_ = 42	Counseling using Mother's Card with messages on promoting effective play and communication between a caregiver and child with demonstration, practice, and problem-solving based on the WHO program.	Home visits	2 home visits: once at the baseline assessment and once within 6 months of the first counseling session	Health professionals	Unclear
Luoto (56)	Group-only model (*N* = 346) Mixed-delivery model (*N* = 373) Control group (*N* = 351)	Msingi Bora (Good Foundation) curriculum adapted from previous parenting programs in LMICs. It involved 6 sessions with messages on 5 key practices: responsive play, responsive communication, hygiene, nutrition and love and respect in the family	Group sessions & Home visits	Group-only model involved 16 group sessions delivered fortnightly. Mixed-delivery involved 16 group sessions delivered fortnightly and 4 home visits	Community health volunteers	No intervention
Muhoozi (42)	Intervention vs. Control N_int_ = 243 N_ctrl_ = 224	A nutrition education program on complementary feeding plus messages on hygiene, sanitation, and play	Group sessions	3 main group sessions each lasting 6–8 h supplemented by monthly group meetings & home visits to encourage practice and adherence across 6 months	An education team of 4 trained persons with bachelor degrees in nutrition & village health team leader or mother leader	Unclear
Murray (58)	Intervention vs. Control N_int_ = 127 N_ctrl_ = 136	Counseling mothers in sensitive and responsive interactions with her infant based on principles of the WHO program and another program plus the regular visits (see details in the control arm).	Home visits	16 home visits: twice during the 3rd trimester of pregnancy, weekly for 2 months postpartum, fortnightly for 2 months, and monthly for 2 months across 8 months	Mothers from the community	Fortnightly visits by community health workers monitoring maternal and infant health.
Nahar (44)	Stimulation (PS) vs. no stimulation (CC, & CH) N_int_ = 59 N_ctrl_ = 118	Play sessions with low-cost toys with mother-child pairs and parental education on child development, the importance of play, chatting and praising the child with demonstration on incorporating play into daily activities plus routine care (see details in the control arm).	Individual visits at the clinic	9–12 visits: fortnightly for 3 months and fortnightly (35% of the sample) to monthly for 3 months across 6 months	Female health workers with 8–10 years of education	Routine care at the clinic on growth monitoring, health education, micronutrient supplementation, and other health care.
Powell (51)	Intervention vs. Control N_int_ = 65 N_ctrl_ =64	Demonstrate age appropriate play activities using home-made toys and books and discuss parenting issues, including the importance of praise, attention, and responsiveness, appropriate discipline strategies, child nutrition, and ways to promote children's play and learning.	Home visits	50 weekly home visits for 12 months	Community health aides	Unclear
Rockers (49)	Intervention vs. Control N_int_ = 220 N_ctrl_ =215	Group meeting on cognitive stimulation and play practices, child nutrition and cooking practices, and self-care for good mental health; home visits on health monitoring and counseling on using health services.	Group sessions & home visits	24 fortnightly home visits on health and nutrition & 20 fortnightly group meetings on stimulation for 12 months	Child Development Agents for home visits & head mothers for group sessions	Routine care
Singla (46)	Intervention vs. Control N_int_ = 160 N_ctrl_ =131	Five messages related to child care (play, talk, diet, hygiene, and love and respect) were delivered to groups of parents through demonstration, practice, and role-playing activities. Maternal well-being and father's involvement were also discussed in separate sessions.	Group sessions	12 group sessions fortnightly over 6 months & 1–2 home visits to review the messages & 1 booster session 2 months after intervention	Community health workers	Received nutrition information at the end of the baseline interview
Tofail (45)	Stimulated vs. Unstimulated *combined IDA and NIDA groups* N_int_ = 224 N_ctrl_ = 210	Activities for mothers on how to play with toys and interact with their children in a way to promote their development modified from the Jamaica program. IDA group received iron syrup for the first 6 months.	Home visits	36 weekly home visits for 9 months	Female play leaders from the village with 9–12 years of education	Visited weekly on monitoring child's health status
Vazir (47)	Stimulation (RCF & PG) vs. Control (CG) N_int_ = 153 N_ctrl_ =182	Mothers received 11 messages on complementary feeding, 8 messages on responsive feeding, and 8 messages and activities on stimulation using five simple toys over and above the routine services.	Home visits	30 home visits: twice per month for 3 months, 4 times per month for 3 months, and twice per month for 6 months across 12 months	Women from the village with high school education	Routine services on breastfeeding and complementary feeding, growth monitoring, and provision of supplemental food.
Walker (52)	Intervention vs. Control N_int_ = 63 N_ctrl_ = 68	Mothers received training on having conversations with the infant, responding to infant's cues, showing affection, and focusing infant's attention on the environment.	Home visits	8 weekly home visits for two months	Community health worker	unclear
Yousafzai (39)	Stimulation (w/o nutrition) vs. no stimulation (nutrition and control) N_int_ = 701 N_ctrl_ = 680	Caregivers received training on sensitivity and responsiveness through developmentally appropriate play activities. Enhanced nutrition provides knowledge about the associations between good nutrition and health, additional advice on responsive feeding, and development of counseling skills and problem solving about feeding and micronutrient powder.	Group sessions & home visits	24 monthly home visits & 24 monthly group sessions for 24 months	lady health workers	Lady health workers provided standard-of-care services on health, hygiene, and basic nutrition education.

#### Mode and Dose of Intervention Delivery

Interventions were delivered through home visits in nine studies and group sessions (with or without home visits) in 12 studies. Of note, 71% of the studies in the rural context used group sessions compared to 29% of the urban or peri-urban studies.

The interventions were delivered in varying levels of frequency, ranging from twice weekly to two times over the course of 6 months. The average frequency was two times or less per month in nine studies and over twice per month in 12 studies. The total number of sessions ranged from 2 to 124, with the majority of the interventions (15 out of 21) delivered in 12 or more sessions. The overall duration of the interventions ranged from 2 to 24 months, with the majority (14 out of 21) under 12 months.

#### Delivery Agents

Ten studies (39, 40, 43–47, 49, 58, 59) clearly reported that the intervention delivery agents were women; however, gender was not clearly stated in some studies (41, 50–53, 55). Only eight of the 21 interventions relied on trained delivery agents who did not formally work with the local health care system (e.g., women who were selected to serve as play leaders, volunteers, or home visitors based on being mothers, having a reputation in the community, or having some basic education and good communication skills) (40, 43, 45–47, 55, 57, 58).

All studies reported that intervention delivery agents were trained, with the majority indicating that the delivery agents received one-off in-person training sessions ranging in length from 3 days of training (41, 55) to 3 weeks of training (40, 45, 58). Overall, in 10 intervention studies the delivery agents' training duration was 2 weeks or more (40, 43, 45, 46, 51, 52, 56–59); however, the training duration was not clearly reported in 5 studies (42, 44, 47, 50, 53). Refresher training of delivery agents was reported among four intervention studies (46, 54, 56, 61). In some studies, training sessions were spread out within the implementation period (40, 56, 58). Depending on the specific focus of the intervention, general training content included theoretical and practical skills on counseling and effective communication (e.g., facilitating support groups, problem identification and solving) and parenting practices (including responsive feeding, child stimulation, play). Trainers included trained graduates (mainly in health sciences like nutrition health and psychology) (40, 42, 59), trained paraprofessionals (49), professionals in ECD (61), researchers together with staff from implementing/local organizations working in child and maternal health (46, 54, 56). However, the majority of the studies did not report in detail on training aspects.

#### Intervention Curricula

There were various reported sources of intervention curricula, with a common tendency for studies to report multiple sources. Some of the commonly cited sources included UNICEF and WHO's “Care for Child Development” program (39, 49, 57), the Jamaica Home Visiting program (40, 59), WHO/PAHO guidelines on complementary feeding (42, 47, 54), WHO's social Baby and Improving psychosocial development (58), among others. However, in some of the studies, the sources of the curricula were not clearly reported (55, 56). Routine care, mostly referring to routine services provided mainly by community healthcare systems, was the most typical reported component in the control groups.

#### Fidelity and Quality Control

Various modalities were utilized to ensure fidelity and quality of the implementation process (See Supplementary Table 2). Some commonly reported ones included: routine on-the-job observation of agents by supervisors (mostly on a monthly basis), utilization of designed monitoring tools such as visit forms, fidelity checklists and home visit forms to track progress and compliance to curricula (41, 49, 54); formal and informal sessions involving supervisors and delivery agents were utilized to provide feedback and to discuss challenges and solutions to keep activities on track (41, 44, 52, 56). Although the majority of studies reported that delivery agents were provided with support supervision, most did not elaborate on what such supervision entailed. A few indicated that it entailed assistance of the agents to prepare their sessions, provision of feedback on performance through a structured process (e.g., monitoring forms), reviewing the intervention topics with them and providing them guidance in discussions and practice prior to their activities (41, 46). Other quality improvement approaches reported feedback on the intervention experience directly from the beneficiaries (47), providing mentorship and on-the-job coaching for delivery agents (40, 59), and sending frequent reminders reinforcing key messages to delivery agents (40).

#### Moderation Effects

Effect sizes at each level of the moderators are summarized in Table 4. These effect sizes are for individual moderators from un-adjusted moderation analyses and thus should be interpreted cautiously. Moderations were not reported for social-emotional outcomes due to the small number of studies reporting on this domain. Generally, 5 or more studies are needed to reasonably and consistently achieve sufficient power from random-effects meta-analyses (62).

**Table 4:** Effect sizes by moderators.

**Study**	**Cognitive**			**Language**			**Motor**		
	***n***	***M*_**ES**_ [95% CI]**	***Q*_**between**_ & *p*-value**	***n***	***M*_**ES**_ [95% CI]**	***Q*_**between**_ & *p*-value**	***n***	***M*_**ES**_ [95% CI]**	***Q*_**between**_ & *p*-value**
**Context of the sample**			Q = 1.48, *p* = 0.22			Q = 1.69, *p* = 0.19			**Q = 5.20**, ***p*** **= 0.02**
Rural	11	0.48 [0.29, 0.67]		8	0.40 [0.21, 0.59]		8	0.29 [0.17, 0.41]	
Urban/Peri-Urban	7	0.34 [0.19, 0.46]		5	0.20 [-0.04, 0.44]		6	0.11 [0.02, 0.19]	
**Child age at enrolment**			Q = 1.3, *p* = 0.25			Q = 0.14, *p* = 0.71			Q = 2.60, *p* = 0.11
<12 months	9	0.49 [0.24, 0.75]		6	0.33 [0.03, 0.63]		7	0.27 [0.11, 0.44]	
≥12 months	8	0.33 [0.23, 0.43]		6	0.27 [0.12, 0.42]		6	0.10 [-0.03, 0.23]	
**Maternal education**			Q = 1.32, *p* = 0.25			**Q = 4.25**, ***p*** **= 0.04**			Q = 2.29, *p* = 0.13
< primary	11	0.48 [0.28, 0.67]		7	0.45 [0.25, 0.64]		9	0.25 [0.13, 0.38]	
≥ primary	6	0.34 [0.20, 0.47]		5	0.17 [−0.01, 0.35]		4	0.12 [0.01, 0.23]	
**Mode of delivery**			**Q = 4.99**, ***p*** **= 0.03**			Q = 2.69, *p* = 0.10			**Q = 7.59**, ***p*** **= 0.006**
Individual visits alone	8	0.28 [0.19, 0.36]		4	0.17 [0.004, 0.33]		7	0.08 [−0.02, 0.18]	
Group sessions or group together with individual	10	0.53 [0.33, 0.73]		9	0.38 [0.19, 0.57]		7	0.31 [0.19, 0.44]	
**Dose of delivery**			Q = 0.12, *p* = 0.73			Q = 0.12, *p* = 0.73			Q = 0.01, *p* = 0.94
≤ 2 times/month	9	0.46 [0.19, 0.73]		6	0.31 [0.02, 0.59]		5	0.20 [−0.04, 0.44]	
> 2 times/month	9	0.41 [0.29, 0.52]		7	0.37 [0.20, 0.54]		9	0.21 [0.08, 0.34]	
**Number of sessions**			Q = 0.03, *p* = 0.81			**Q = 6.24**, ***p*** **= 0.01**			Q = 0.02, *p* = 0.89
<12	5	0.42 [0.28, 0.56]		5	0.12 [−0.04, 0.28]		5	0.22 [−0.04, 0.48]	
≥12	13	0.44 [0.27, 0.62]		8	0.44 [0.25, 0.63]		9	0.20 [0.07, 0.33]	
**Duration of intervention**			Q = 0.37, *p* = 0.54			Q = 0.50, *p* = 0.48			Q = 0.02, *p* = 0.89
<12 months	12	0.46 [0.27, 0.66]		10	0.30 [0.10, 0.50]		8	0.20 [0.03, 0.36]	
≥12 months	6	0.37 [0.19, 0.56]		3	0.43 [0.13, 0.74]		6	0.21 [0.05, 0.38]	
**Delivery agents**			Q = 2.72, *p* = 0.26			Q = 0.02, *p* = 0.99			Q = 0.51, *p* = 0.77
Trained Paraprofessionals with previous experience in the healthcare system	6	0.46 [0.31, 0.60]		5	0.33 [0.08, 0.58]		5	0.15 [−0.14, 0.43]	
Trained Paraprofessionals without previous experience in the healthcare system	7	0.32 [0.23, 0.43]		3	0.32 [0.05, 0.59]		5	0.18 [0.05, 0.32]	
Mixed Paraprofessionals and/or health professionals	5	0.58 [0.08, 1.08]		5	0.35 [−0.04, 0.75]		4	0.27 [0.02, 0.52]	
**Duration of training of delivery agents**			Q = 3.29, *p* = 0.19			Q = 2.3, *p* = 0.31			Q = 2.3, *p* = 0.31
2 weeks or more	8	0.33 [0.23, 0.43]		5	0.23 [0.06, 0.40]		5	0.13 [0.03, 0.23]	
Less than 2 weeks	5	0.57 [0.22, 0.92]		5	0.45 [0.21, 0.70]		4	0.27 [0.11, 0.43]	
Details not reported	5	0.45 [0.29, 0.57]		3	0.33 [0.18, 0.50]		5	0.15 [−0.16, 0.46]	

*Bolded results indicate that there is statistically significant variation in effect size across the groups*.

Among the sample and context characteristics [i.e., study setting (rural and urban/peri-urban), child age at enrolment and maternal education], we found statistically significant group differences in the pooled effect sizes across maternal education (for language skills) and rural settings (for motor skills). Specifically, the effect sizes for language were larger for children of caregivers with lower educational level (ES = 0.45 vs. 0.17, Q = 4.25, *p* = 0.04). The context where the intervention was delivered (urban vs. rural), as well as the mode of delivery, also explained a significant proportion of variance on the effect sizes on motor skills outcomes. Effect sizes were greater for children in rural areas compared to urban settings (ES = 0.29 vs. 0.11, Q = 5.2, *p* = 0.02). Thus, consistent with previous research, we find that program effects were larger for vulnerable children, including lower levels of family education and rural residence.

The moderation effects of the intervention characteristics of mode of delivery, duration of the intervention, number of sessions delivered, dose of delivery, the type of delivery agents as well as the duration of their training are also summarized in Table 4. Statistically significant group differences in the pooled effect sizes were only found across the mode of delivery (for cognitive and motor skills) and the number of sessions (for language skills). The effect sizes for language skills were larger for children of caregivers who received interventions with 12 or more sessions (ES = 0.44 vs. 0.12, Q = 6.24, *p* = 0.01). Effect sizes for motor skills were also greater for interventions delivered through group sessions compared to individual sessions (ES = 0.31 vs. 0.08, Q = 7.59, *p* = 0.006). Also, for the cognitive development domain, the effect sizes were greater for interventions delivered through group sessions compared to individual sessions (ES = 0.53 vs. 0.28, Q = 4.99, *p* = 0.03).

## Discussion

This systematic literature review identified 21 parenting interventions published from 2004 to 2020 with the components of responsive, stimulating, and sensitive caregiver-child interactions for children under age two in low- and middle-income countries evaluated in randomized controlled trials. We also summarized the various intervention features and implementation aspects, for example, relating to context and participant characteristics, quality and fidelity, capacity building, delivery agents, and intervention curricula. We investigated how some of these features moderated the effects of these interventions on childhood developmental outcomes.

A key finding from our meta-analyses is that there are overall positive, albeit small-to-medium, effects of the parenting interventions on childhood developmental outcomes, including cognitive, language, and motor skills. Our findings are consistent with findings from a previous systematic review published in 2015, where stimulation interventions among children aged 0–2 years were moderately effective for cognitive outcomes (d = 0.42) and language development (d = 0.47) (21). Similarly, our results are supported by findings from a more recent systematic review that reported significant effect sizes of 0.49 in cognitive skills, 0.43 in language skills, and 0.39 in motor skills among interventions with components on responsive care and learning opportunities LMICs (17). In our present review, we presume that the lack of statistical significance for the socio-emotional domain may possibly arise from the few intervention studies (*n* = 7) that were currently published and eligible. Taken together, our updated review captures literature up to the end of 2020 and shows that results on intervention effects in the cognitive, language and motor skills domains are, more or less, consistent with previous systematic reviews in this area.

Although cognitive-linguistic development in the early years is important in relation to later educational achievement, the review supports our call for increased attention to social-emotional outcomes for three main reasons. First, early social-emotional development plays a key role in subsequent achievement, health, and well-being over and above the contribution of early cognitive development, especially in adverse contexts (63–66). Second, social-emotional development is sensitive to influences of early adverse experiences such as exposure to violence and stress (7, 67–70). Third, social-emotional development can be crucially shaped through parenting techniques such as developmentally appropriate support and warmth, engaging in sensitive parent-child interactions, and fostering secure parent-child bonds (71–74).

Another important finding is the significant variation in the overall effect of the interventions on the domains of cognitive, language and motor skills that were attributable to some intervention and implementation features. First, the use of group sessions or group sessions combined with home visits in our review was found to be more beneficial than individual sessions alone (specifically for cognitive and motor skills development). This approach has been endorsed by other researchers who suggest that its effectiveness arises from its potential to modify group norms on child-rearing, being less labor intensive compared to home visits and because it encourages peer support (21). However, it is unclear why the variation in effects attributable to delivery modality was only registered in cognitive and motor development and not in the language domain.

Still, in connection to the delivery approach, we found that interventions that delivered a greater number of sessions (12 or more) across the intervention period were more beneficial for language development compared to those with fewer sessions. Studies on the dose-effect of interventions on language development are scanty; however, one systematic review found that interventions aimed at improving child language through enhancing maternal responsivity were generally effective when offered for ~10–12 weekly sessions programs (75). We suggest that the findings indicating better effects of the interventions among children of rural (for motor skills) and lower educated (for language skills) caregivers are symbolic of catch-up neurodevelopmental growth in children from less privileged settings where social inequalities and a myriad of negative social determinants of health hinder optimal neuro-development in the early years (76). Another explanation may be that the differences result from the smaller number of studies conducted in urban settings and higher educated caregivers.

The findings on the use of formally trained paraprofessionals (e.g., community health volunteers) as intervention delivery agents accentuate their central role in promoting community health and early child development (77), but more importantly, the feasibility and promising sustainability of utilizing this delivery approach for mainstreaming nurturing care in community health within LMIC setting. Another important finding was that some interventions were still feasible and effective even when trained community resource persons who were not necessarily trained formal paraprofessionals and without prior work experience in the healthcare system were utilized as delivery agents. This is important because formally trained health paraprofessionals may sometimes face large workloads from their formal roles and various ongoing community health program activities (78, 79) that might negatively impact their performance when asked to offer extra duties on parenting interventions. However, owing to their formal recognition in the communities and by the governmental health structures, their extensive experience and accrued training in the cross-cutting aspects of child and maternal health (77, 80), formally trained health paraprofessionals like community health volunteers are likely to present a more sustainable choice of delivery agents of parenting interventions.

Our findings on capacity building and fidelity/quality control measures utilized among the interventions have important implications. First, current studies on parenting interventions inconsistently report about core attributes (content, methods, duration, trainers) of training programs for intervention delivery agents. Although there is likely to be extensive variation in training content and duration, partly due to previous formal training possessed by the paraprofessionals, it is important to have some basic/foundational skills and knowledge areas (modules) for improved scalability and quality assurance of parenting interventions across settings. We also argue that the brevity of the training (most without refresher training) of delivery agents in many of the interventions was because most of these were brief interventions lasting less than 12 months. Second, a number of useful and transferable quality assurance techniques can be borrowed for future interventions. Notable among these is the process monitoring using designed monitoring tools like fidelity checklists, support supervision and on-the-job mentoring, maintaining ongoing open feedback and process learning, and involving beneficiaries in appraising the implementation process. Third, the current evidence indicates the need for more implementation research and better data to determine which features (i.e., training, skills, supervision, remuneration) predict program effectiveness and efficiency. Besides, clearer documentation of the formative and implementation aspects, including the sources of intervention components and the curriculum/intervention adaptation procedures, are crucial for improving scalability and adaptation to new settings and the comparability of study findings across different settings.

The major strengths of this systematic review include the expansion of the existing body of evidence on stimulation and responsive caregiving interventions in LMICs by including the most recent studies published in this area, expansion of the domains to include domains of social-emotional development and the thorough synthesis of the intervention and implementation features, including quantitative analysis of the moderation effects of various intervention features on the intervention effect sizes on childhood development outcomes. Our findings should be interpreted cautiously due to several limitations that should be noted. First, we only included a small body of randomized controlled trials and thus excluded interventions that were evaluated in other less rigorous designs (e.g., quasi-experimental) and those that did not assess child developmental outcomes. Nonetheless, the excluded interventions may still contribute valuable information to the field (22, 81). Second, the overlap in the classification of the sub-categories of the moderators presents challenges in delineating the specific impact of the moderator factor on the effectiveness of the intervention. For example, although the number of sessions (<12 vs. ≥12), age of the child at enrolment (<12 months vs. ≥12 months), study context (Rural vs. Urban/Peri-Urban), dosage (≤ 2 times/month vs. > 2 times/month), duration of training for delivery agents, among other factors, are classified into simplified binary or categorical variables, there are variations within these sub-categorizations which can have varying moderation on the overall effectiveness of the interventions. Nonetheless, such broader categorizations, as in the case of our analysis, are feasible given the limited number of studies currently available within this field. Although we conducted meta-analysis only for neurodevelopmental outcomes with 5 or more studies, we found that for the moderation analysis, some of the sub-categories of the moderator variables had fewer than 5 studies. This was specific for the moderators on delivery agents in both motor and language sub-domains, for duration of the intervention (in the language domain) and maternal education (in the motor domain). This may potentially attribute to the statistical non-significance of these moderators. Besides, there are additional factors that are theoretically important in explaining the heterogenous effect sizes but could not be evaluated with the limited evidence base (82). Examples include the timing (e.g., from birth vs. 12 months), the level of adaptation of intervention curriculums and assessment tools to the local context, and the quality of implementation (e.g., fidelity, training, supervision). Nonetheless, we have narratively summarized in more detail some of these implementation features and draw lessons for future research. Third, we did not conduct meta-regression to assess the unique effect of each moderator on intervention effect sizes while controlling for other moderators, owing to a small number of studies. Instead, we computed effect sizes separately for each moderator and therefore, the estimates should be interpreted with caution because overlap among the moderators was not accounted for. It is plausible that in an adjusted moderation analysis (i.e., when more than one moderator is included in the same model), statistical significance may shift and become attributable to other moderating factors than those currently identified in the un-adjusted analyses. Therefore, in the future where more studies are available in this field, adjusted meta-regression will be more informative in elucidating the implication of intervention characteristics on the effectiveness of parenting interventions with components of psychosocial stimulation or responsive caregiving on cognitive, language, motor and socio-emotional skills development of children.

## Conclusion

Notwithstanding the limitations noted above, this review provided a valuable synthesis of the evidence on parenting interventions for children under age two in low- and middle-income countries. In spite of the promising effects, this is still a small area with significant potential to grow. We noted several directions that warrant further investigation. First, inadequate nurturing care often co-occurs with other risks in the environment, such as maternal mental health problems, lack of social support, and exposure to violence within the family and community. It may be beneficial to explore integrative models that combine nurturing care with other interventions that address environmental risk factors. One example of such models comes from rural Uganda, where researchers incorporated modules promoting paternal involvement and maternal mental health into a nurturing care intervention (46). Second, in spite of the converging evidence for short-term benefits on early child development, there is still a dearth of follow-up studies examining the extent to which demonstrated effects sustain later in development. Evidence from a small-scale intervention of stunted 9- to 24-month children in Jamaica suggested promising long-term benefits on educational, social-emotional, and economic outcomes at 22 years of age (22, 83, 84). In contrast, follow-up studies from two more recent RCT trials of children from rural Pakistan (39, 85) and urban Colombia (40, 86) produced mixed findings on whether the intervention effects are sustained 2 years after the intervention. Clearly, more research is needed to better understand the long-term benefits of early interventions and whether booster interventions at a later stage are needed. Third, despite the overall positive effect for children who received early interventions, the mechanisms (i.e., why the intervention works) and specificities (i.e., in what contexts and for whom the intervention works more or less) the effectiveness are still not well-understood. Only a small number of studies examined the mechanisms (e.g., maternal sensitivity, home stimulation) through which the intervention had an impact on child outcomes (46, 87, 88). Researchers have just begun to explore child and family characteristics (e.g., biological sensitivity, socioeconomic status) that may account for the differential intervention effects (58, 89). In light of this, we call for more investigations in this arena using existing data and future research.

Finally, nurturing care interventions in LMICs have been mainly implemented in low-income populations and populations at risk for undernutrition and rarely in other adverse contexts, such as those affected by armed conflicts and forced migration. In recent years, researchers have brought attention to the value of nurturing care interventions in fragile and conflict contexts and their potential to reduce violence and promote peace (4, 90). The Early Childhood Peace Consortium is one such innovative approach aimed at peacebuilding through investment in early child development (91). Although this work is still in the infancy stage, preliminary evidence has shown a promising effect on violence reduction and child development (9, 92). With an eye toward the future, more research with more detailed documentation of the intervention and implementation features is needed to understand what works, what contexts, and why in early parenting interventions.

## Data Availability Statement

The original contributions presented in the study are included in the article/Supplementary Material, further inquiries can be directed to the corresponding author/s.

## Author Contributions

The idea of this review was envisaged by KM, M-CM, TM, and SL. LZ and DS developed and ran the search strategies, screened the titles and abstracts, as well as the full texts, and assessed the methodological quality. LZ, TM, KP, M-CM, AA, and GM developed the protocol. LZ, DS, and JM extracted data. KP, M-CM, AA, TM, GM, KM, and SL provided technical advice. LZ and DS drafted the manuscript with input from KP, M-CM, AA, KM, and TM. All authors have contributed significantly by taking part in reading, writing, and reviewing this manuscript.

## Conflict of Interest

The authors declare that the research was conducted in the absence of any commercial or financial relationships that could be construed as a potential conflict of interest.
