# Supplementary material for: Supporting Child Development Through Parenting Interventions in Low- to Middle-Income Countries: An Updated Systematic Review
Source: Front Public Health. 2021 Jul 16;9:671988. doi: 10.3389/fpubh.2021.671988 (PMC8322584; doi:10.3389/fpubh.2021.671988)
Supplement: Supplementary file 1 [file Table_1.DOCX]

**Supplementary materials:**

**Table S1: Literature Search Keywords and Databases**

| Search Strategy | Content |
| --- | --- |
| Databases | PubMed, EMBASE, CINAHL, PsycInfo, Scopus, ERIC, ProQuest Dissertation & Theses Global, EconLit, OAIster, OpenGrey |
| Keywords | *Type of Participants:*  (child* OR toddler* OR infant* OR infancy* OR baby* OR babies* OR newborn* OR neonatal*)  AND  *Type of Interventions:*  (responsive* OR caregiver* OR parent* OR attachment* OR stimulation* OR childrearing* OR child-rearing* OR psychosocial* OR psycho-social*) & (intervention* OR program* OR group* OR trial* OR RCT* OR education*)  AND  *Type of Outcomes:*  (development* OR cognitive* OR cognition* OR language* OR literacy* OR vocabulary* OR communication* OR social* OR emotion* OR behavior* OR behaviour* OR socio-emotion* OR mental* OR motor*) |
| Date range | 2015 to date of search (12/2020) |
| Language | English |

**Table S2: Additional implementation characteristics**

| **Author** | **Delivery agent** | **Experience [qualities agents]** | **Capacity building/Training** | **Quality control** | **Formative research** |
| --- | --- | --- | --- | --- | --- |
| Gardner (50) | Community Health Workers (CHWs) | Details not provided | CHWs were specially trained (study does not provide details) | Observation of intervention activities by a supervisor [monthly about 5 interviews] | Curriculum was culturally appropriate and previously used in studies with Jamaican mothers and children |
| Muhoozi (42) | Trained graduates  Village Health Team (VHT) leaders or mother leaders | Trained graduates: Bachelor’s degree in Nutrition.  VHT/mother leaders:  Selected by consensus | VHT leader/mother leaders attended all the 3 main education sessions (delivered by the trained graduates) | Observation of VHT’s intervention activities by study team.  Follow-up with mothers in homes to encourage adherence to intervention    Assessment based on reports and interactions with VHTs | Curriculum was based on the 10 guiding principles of complementary feeding (PAHO/WHO, 2003) and the Stimulation component based on socio-cognitive learning theory.  Curriculum adaption processes not mentioned |
| Nahar (44) | Female trained health workers (play leaders) | 8-10 years of schooling | Health workers were trained (study does not provide details) | Feedback and debriefing.  Observation of intervention activities by a supervisor [monthly] | The study protocol was changed in the course of the study to increase the frequency of visits as loss-to-follow up was high (about 37%) |
| Powell (51) | Community Health aides | Paraprofessionals, assist in clinics and visit homes to give advice on health and nutrition  Had 6-8 weeks’ training, primarily in maternal and child health | Attended 2 additional one-week workshops covering child development and the intervention | Observation of intervention activities by a supervisor [monthly]  Feedback and records review: | Piloting clinic-based versus home-based sessions    The curriculum was previously used in studies within same context but was further adapted for this study so that fewer tools and materials were utilized |
| Rockers (49) | Trained community-based health worker (referred to as Child development agents (CDAs)  Local ‘head mother’ | Trained community-based health worker (CDA):  At least a 9th grade education.  Had previous experience in providing community-based health services as part of the formal health system.  Local head mothers:  selected by the community by consensus | CDAs: underwent a 5-day training during which they received a refresher on diagnosing child illness and supporting child development, learnt the protocols for the home visits and were trained on how to facilitate and support the group meetings  Head mothers: Were provided training and resources by the CDAs prior to each round meeting in accordance with the curriculum for the specific meeting round. | Minimizing caregiver burden: Few meetings in locations chosen by the community. Meetings combined locations to minimize the travel burden  Record keeping on progress: CDAs completed visit forms which provided key information to track the implementation | Consultations on caregiver involvement  Curriculum content borrowed from existing child development programs e.g. Care for Child Development (WHO/UNICEF) and Inter-Agency Taskforce on HIV & ECD. |
| Vazir (47) | Village Women (VW) | Village women (VW):  Were mothers and had a high school education | The VW received supervised training on how to counsel caregivers using the pictorial flip charts.  The VWs visiting the 2 groups of the intervention arm were trained separately with specific flip charts | Feedback and records review.  Trained graduates in nutrition supervised the VW, examined their records of visits and asked mothers independently what they were told in the VWs’ last visit | 6 months prior to the sample selection, 4 villages from the same district were involved in formative research to understand the knowledge and beliefs, motivations and aspirations of mothers about child feeding practices, and child stimulation.  Photographs modelling specific behaviors were produced as part of the formative research.  Messages were pre-tested to assess feasibility of adoption considering availability and affordability  PAHO/WHO guidelines on complementary feeding were used for designing messages on complementary feeding. |
| Murray (58) | Home visitors | Home visitors: were mothers themselves. 2 had completed schooling; none had education or training beyond school | Home visitors received 3 weeks training on the intervention over a period of 4 months | Supervision: received weekly group supervision from a community clinical psychologist | Curriculum content based on principles of WHO’s Social Baby and Improving the psychosocial development of children.    Curriculum adaption processes not mentioned. |
| Tofail (45) | Play leaders (PLs) | PL: women who had completed 9 to 12 y of education and were living in the villages | 3 weeks of training in home visiting techniques and the curriculum | Supervision: 3 supervisors mentored the Play leaders  Observation of intervention activities by a supervisor [monthly] | The curriculum was previously used in Jamaica but was modified to be culturally appropriate for Bangladesh. |
| Walker (52) | Community Health workers | No details provided | 2 weeks of training in child development, the conduct of the intervention, and on the use of the curriculum manuals. During the training, the CHWs also assisted with the preparation of the toys used in the intervention | Observation of intervention activities by a supervisor [monthly].  Feedback and debriefing. | No details reported |
| Hamadani (43) | Play leaders | Play leaders: literate women trained for 2 weeks | Play leaders were trained for 2 weeks (study does not provide details) | Supervision: 1 of 2 supervisors attended the visits conducted by play leaders regularly | The curriculum was based on one that was previously used in Jamaica and modified for use in Bangladesh  Initially, focus-group discussions were conducted with mothers of undernourished and well-nourished children to determine their practices, knowledge, and attitudes toward child development. |
| Aboud (48) | Community leaders  Government paid family welfare assistants | Community leaders:  Young women with 10th grade education  Recommended by community leaders  Family welfare assistants:  =Formally hired by the government  =Had 12^th^ grade training & many years of experience  =Received training through a government model including messages about feeding and hygiene | Community leaders:  4 days of training (by organization) spread-out in 4 months. Focused on understanding the benefits of the parenting practices and demonstrating when and how to do them, along with communication skills for leading a group session.  A 3 bi-monthly 1-day refresher trainings devoted to reviewing topics and solving problems  Family welfare assistants:  An initial 2-day course.  A mid-program refresher day. The training focused on how and why parents were to practice the messages. | Record keeping on fidelity: a fidelity checklist was used to document if facilitator demonstrated activities with a child, used available tools and materials during the session.  Observation of intervention activities by a supervisor [during first 4 months]. | The intervention components borrowed from WHO guidelines on complementary feeding, Psychosocial development of young children and from the social learning theories. Also prom previous programs implemented in Bangladesh. |
| Aboud (54) | Local community health workers  Peer educators | Local community health workers  Peer educators:  Young women from villages with a grade 9 education | Peer educators: Trained for over 4 days to use a 30 page manual for conducting responsive feeding and play sessions | Observation of intervention activities by a supervisor (irregular visits) to confirm fidelity to the manual. | Not reported |
| Singla (46) | Community volunteers | Community volunteers were selected by the community and Plan Uganda staff on the basis of 3 criteria: their reputation in the community, communication and language skills, and a minimum of sixth grade education | Trained for 14 days during the start and in the middle of the intervention. Training focused on the programme content and effective communication skills | Supervision: entailed weekly supervision involving assistance of community volunteers to prepare their session, discussion of problems encountered in previous sessions, and provision of feedback through a structured monitoring form | The programme was based on formative research done in 2012 to identify parent goals and practices |
| Jin (53) | Health professionals | Health professionals (Health counselors) | Health professionals participated in a pilot-testing course in World Health Organization’s Care for Development counseling materials conducted as part of China’s National IMCI activities. | Not reported | The counseling cards were adapted for China from WHO’s global Care for Development counseling materials. |
| Chang (41) | Community Health Workers  Nurses | Community health workers:  Had 3 or more years’ secondary-level education  Had preservice training of up to 20 weeks or in-service training  Professionals (Nurses) | Training of the CHWs: comprised 3-day workshops with viewing of films and role play | Support supervision: Before a new set of topics was shown, a supervisor visited the clinic, reviewed the topics with the CHWs, and provided guidance in discussions and practice.  Observation, Feedback and debriefing: Supervisor monitored implementation quality every 6 weeks using 3-point ratings of how well the CHW involved the mothers and acknowledged and praised their efforts. | Not reported |
| Attanasio (40) | Home visitors | Selected from among the mother leaders in each of the municipalities.  Hired part-time. | Home visitors were trained for 2 weeks. An additional week of training 1-2 months after the program began.  The training was conducted by mentors with an undergraduate degree in psychology or social work—or fieldwork experience who underwent six weeks’ pre-service training on the home visiting curriculum and protocols, training and supervision skills | Reminder and reinforcement of delivery agents: mentors sent short text messages to home visitors every month, which reinforced key advice. Home visitors were also encouraged to call mentors for advice where necessary.  Observation of intervention activities by mentors [once in 7-10 weeks] to monitor the fidelity of the implementation, provide support. | The Jamaican Home Visiting model was the basis for the psychosocial stimulation component and was adapted to suit the Colombian socio-cultural context (e.g. curriculum and materials) |
| Yousafzai (39) | Lady Health Workers (LHWs) | Women between 18-45 years.  At least 8 years of education who are trained over 15 months and 12 months field-based training.  Part of the local healthcare system and involved in maternal and child health, primary health care, health education etc | LHWs received 2 days of basic training on nutrition.    The LHWs received 3 days of basic training on responsive stimulation and delivery was integrated into existing routine home visits.  LHWs who provided the combined intervention received 5 days of basic training, received 1 refresher training day every 6 months and on-the-job coaching every month.  Training was provided by a team of ECD facilitators. | Supervision: A team of early child development supervised the LHWs. | The intervention was based on the adaptation of the Care for Child Development Package of UNICEF and WHO. |
| Abimpaye (55) | Local volunteers | Local volunteers | In the light touch, local volunteers received three half-day trainings and a basic package of training materials.  In the full intervention, local volunteers received a more robust package of materials, additional training on how to use the materials and were supported by a salaried facilitator | Support supervision: Salaried facilitators supported local volunteers in guiding group sessions and conducting home visits | Not reported |
| Attanasio (59) | FAMI mothers | FAMI mothers:  Mothers with average age=42 years.  Completed 13 years of education.  About 12 years work experience.  No specific training in ECD. | FAMI mothers were trained for three and a half weeks (85 hours)  A team of 9 tutors, with college degrees in psychology and social work, trained and supervised by the research team, trained the FAMI mothers in the intervention | On-the-job coaching and supervision: on the-  job observations and feedback sessions, which took place approximately every 6 weeks | The curriculum borrowed from the Jamaica Home Visiting Intervention model but extensively adapted for use in the FAMI program. New activities like group discussions and language activities were incorporated |
| Barnhart (57) | Community Based Volunteers (CBVs) | CBVs were 18 years of age or older, able to read, write, and count in Kinyarwanda; had to be committed to young children and family values; have sufficient time to carry out intervention training and delivery; and were to be recommended and approved by local community and authorities. | CBVs attended a 2-week training session prior to delivering the intervention.  Supervisors received coaching from trained psychologists who listened to audio recorded sessions and met with the supervisors twice a month during fidelity assessment and strengthening sessions. | CBVs received expert supervision from the bachelor’s level staff members.  Audio-recordings of the intervention delivery by CBVs were reviewed by supervisors who provided feedback (via the phone) on how to improve performance.  Supervisors provided CBVs with in-person shadowing in families’ homes and provided immediate feedback after sessions.  Supervisors facilitated monthly discussions with CBVs to address challenges and support group problem solving. | The adaptation involved input from local and international ECD experts, government stakeholders and local community advisory boards.  Curriculum borrowed from the Family Strengthening Intervention for HIV (FSI-HIV) and WHO/UNICEF Care for Child Development Packages.  An initial pilot study with 20 families was used to refine the final intervention’s curriculum and training materials.  The curriculum was reviewed by counterparts at the Ministry of Gender and Family Promotion, the National Commission for Children, UNICEF and Imbuto Foundation, the lead government agencies and organizations involved in the programme. |
| Luoto (56) | Community Health Volunteers (CHVs) | CHVs were men and women on average 44 years.  Had a minimum of 7 years of education (average 11) and 9 years of CHV experience.  Were paid a monthly stipend for their duties, according to local policy. | CHVs assigned to the intervention received 2 separate (4 months apart) 8 days of intensive training covering sessions 1-8, and 9-16.  Monthly refresher trainings were conducted.  Train-the-trainers modality of training was done. | Delivery sessions were monitored by trained supervisors from the organization who rated the CHVs on skills like facilitating discussions, coaching parents, quality of engagement.  Supervisor feedback was provided at each end of the session.  Local approvals by introducing the project to village leaders was ensured | The curriculum (Msingi Bora) was adapted from previous successful parenting programs in LMICs and involved expansion to include more activities.  Curriculum was translated in Luo, Swahili and English for the CHVs to use efficiently.  Piloting of the 6 sessions was done in 6 villages prior to the intervention and these villages were not included in the main trial. |
